# Naringenin Ameliorated Kidney Injury through Let-7a/TGFBR1 Signaling in Diabetic Nephropathy

**DOI:** 10.1155/2016/8738760

**Published:** 2016-06-30

**Authors:** Ning Yan, Li Wen, Rui Peng, Hongmei Li, Handeng Liu, Huimin Peng, Yan Sun, Tianhui Wu, Lei Chen, Qingrui Duan, Yixuan Sun, Qin Zhou, Lijiang Wei, Zheng Zhang

**Affiliations:** ^1^Molecular Medicine and Cancer Research Center, Chongqing Medical University, Chongqing 400016, China; ^2^Department of Cell Biology and Genetics, Chongqing Medical University, Chongqing 400016, China; ^3^Department of Bioinformatics, Chongqing Medical University, Chongqing 400016, China; ^4^Chongqing Red Cross Hospital, Chongqing 400016, China; ^5^The Second Clinical College, Chongqing Medical University, Chongqing 400016, China; ^6^The First Clinical College, Chongqing Medical University, Chongqing 400016, China

## Abstract

Diabetic nephropathy (DN) is one of the most common complications of diabetes mellitus (DM). However, the exact mechanism is not clearly understood. In this study, our results showed that 24 h urinary protein, kidney index, and glomerular area were decreased, while creatinine clearance ratio was increased in DN rats when the rats were treated with NAR 50 mg/d for 6 weeks. Mesangial cell (MMCs) proliferation was inhibited in the NAR group by 3,(4,5-dimethyl-2-thiazolyl)-2,5-diphenyl-2H-tetrazolium bromide (MTT), and the cell cycle analysis showed that cells stayed in G2 phase in NAR group. And NAR treatment attenuated the deposition of ECM in DN rats and MMCs. Moreover, our data showed that let-7a was downexpressed in both DN rats and MMCs under high glucose condition. Surprisingly, NAR affected the expressions of Col4 and FN through upregulating let-7a in MMCs. In addition, we found that let-7a negatively regulated the expression of transforming growth factor-*β*1 receptor 1 (TGFBR1), and TGFBR1 was required for the let-7a-mediated downregulation of TGF-*β*1/smad signaling. Interestingly, NAR inhibited TGF-*β*1/smads signaling activation by upregulating let-7a. Therefore, our findings indicated that NAR ameliorated kidney injury by regulating let-7a/TGFBR1 signaling.

## 1. Introduction

Diabetic nephropathy (DN) is the leading cause of end-stage renal disease. Mesangial cell proliferation, excessive extracellular matrix (ECM) proteins accumulation, basement membrane thickening, mesangial expansion, and other mesangial region lesions are involved in the renal pathological process, which ultimately lead to glomerular sclerosis. Although hyperglycemia, hyperlipemia, hypertension, blood flow, and inflammation are participated in the pathogenesis of DN, the exact cause of DN remains unclear [1–4].

Naringenin (4,5,7-trihydroxy flavanone, NAR) is a flavanone compound found in citrus fruits, which is rich in the seeds and peels of fruits. Recent researches showed that NAR had many potentially pharmacological effects such as antioxidant, anti-inflammation, anticancer, and antifibrosis [5–8]. Meanwhile, it was reported that NAR possessed antiproliferative activity in lung cancer, colorectal cancer, and leukemia cell and regulated extracellular matrix synthesis in hepatic satellite cell [9–12]. Furthermore, NAR could decrease the blood glucose level in STZ induced diabetic rats [13, 14], suppress macrophage infiltration into adipose tissue in an early phase of high-fat diet-induced obesity [15], and inhibit production of IL-1*β*, IL-6, type IV collagen, fibronectin, TGF-*β*1, and monocyte chemoattractant protein-1 (MCP-1) in the kidney of diabetic mice [16]. However, the mechanism of the proliferation effect of NAR in DN is unknown up to now.

MicroRNA let-7 and its family members have been reported to participate in many diseases including kidney diseases. Let-7a regulated glucose metabolism and insulin synthesis/secretion with type 2 DM by targeting lin28 pathway [17, 18]. Let-7a and let-7d could affect glucose metabolism by downregulating IL-13 in the skeletal muscle of type 2 DM patients [19]. Also, let-7 family members regulated collagen expression in glomerular mesangial cells under diabetic conditions [20]. Furthermore, our previous experiments showed that let-7a was downexpressed in DN patients, and the let-7a-3 promoter hypermethylation and the rs1143770 polymorphism of let-7a-2 were participated in DN [21, 22]. Moreover, let-7a had been reported to modulate ECM deposition in breast and pancreas cells [23–25]. These findings suggested that let-7a may be an important factor in the effects of NAR in DN.

In the present study, we aimed to explore the protective effects of NAR on kidney as well as its effects on let-7a/TGFBR1 signaling in diabetic nephropathy rats and mesangial cell under high glucose condition. This study demonstrated that let-7a and its related pathway-TGF-*β*1/smad signaling formed a negative feedback to inhibit the deposition of ECM by targeting TGFBR1 in DN. Moreover, NAR might repress glomerular mesangial cells proliferation and accumulation of ECM by let-7a/TGFBR1 signaling in DN, and let-7a may be a novel potential target for NAR protecting against diabetic nephropathy.

## 2. Materials and Methods

### 2.1. Animal Experiments

Twenty-five male Sprague-Dawley rats weighting 120 ± 20 g (four weeks old) were provided by the Experimental Animal Center at Chongqing Medical University. The animals were housed in the clean environment under conditions of controlled temperature of 20–25°C and humidity 65~69%. The rats were randomly divided into a control group (CON, *n* = 5) and a DN group (*n* = 20). The control rats were fed with the normal diet, while the rats in diabetic group were fed with the high-sugar-high-fat diet. Five weeks later, diabetic nephropathy was induced by a single intraperitoneal injection of STZ (Sigma, St. Louis, MO, USA) (30 mg/kg) [26]. High blood glucose level of Tail vein (fasting glucose > 16.7 mmol/L) and high urinary total protein level (total protein level in DN group was more than twice higher than the control group) were measured to confirm early DN in rats [27]. Then, the DN rats (*n* = 18) were assigned into two groups: DN rats treated with saline (DN group, *n* = 8) and DN rats treated with NAR (NAR group, *n* = 10). The rats in NAR group were treated with naringenin (Sangon, Shanghai, China) (50 mg·kg^−1^·day^−1^, gavage), while the rats in DN group were treated with the equal volume of normal saline. Among the three groups, blood glucose was measured each week, and 24 h urine was collected every two weeks. Six weeks after NAR treatment, the rats were sacrificed. Heart blood and 24 h urine were collected for creatinine detection. Renal tissues from each rat were disposed as follows: one was immediately frozen in liquid nitrogen, and the other was fixed with 4% paraformaldehyde. All animal experiments were performed in accordance with the protocols approved by Chongqing Medical University Animal Care Committee.

### 2.2. Biochemical and Histology Assays

Blood glucose was measured by Bayer glucometer. 24 h urinary protein was measured by the coomassie brilliant blue (CBB) method. Urinary creatinine (Ucr) and serum creatinine (Scr) were measured by clinical laboratory. Urinary creatinine clearance (Ccr) ratio was calculated using the following equation: Ccr (mL·min^−1^·kg^−1^) = [urinary Cr (mmol/L) × urinary volume (mL)/serum Cr (mmol/L)] × [1/1,440 (min)] [28]. Right kidney weight (KW) and body weight (BW) were assessed to calculate kidney index: KI = KW/BW × 1000. Kidney tissue paraffin sections were subjected to hematoxylin-eosin (HE) staining. The glomerular areas (*μ*m^2^) with 50 fields of view were analyzed by Image-Pro plus 6.0.

### 2.3. Cell Culture

0.1 g NAR powder was dissolved in 1 mL DMSO and then filtrated using 0.22 *μ*m micropore filter, preserved at 4°C for subsequent use. The solution were diluted in DMEM to different working concentration of 100 *μ*mol/L, 200 *μ*mol/L, 400 *μ*mol/L, 600 *μ*mol/L, and 1000 *μ*mol/L. Mouse glomerular mesangial cell (MMC) line (Academy of Sciences, Shanghai, China) was cultured in DMEM with 20% FBS in a humidified atmosphere containing 5% CO_2_ at 37°C. MMCs were maintained in 5 mmol/L glucose plus 20 mmol/L mannitol as the control group (LG). Cells were maintained in 25 mmol/L glucose as the high glucose group (HG), and cells were treated with 25 mmol/L glucose plus NAR as the NAR treatment group (NAR).

293T cells were preserved in our laboratory and grown in DMEM with 10% FBS as previously described.

### 2.4. MTT Assay

MMCs were seeded at a density of 1.0 × 10^4^ cells/well in 96-well plates with NAR treatment. After treated with NAR for 0 h, 12 h, 24 h, and 36 h, 20 *μ*L MTT reagent was added into each corresponding cultured well. Incubate 4 hours in 5% CO_2_ environment at 37°C. The absorbance in 490 nm was measured by the microplate reader (Bio-Rad, USA).

### 2.5. Flow Cytometry Assay

MMCs were treated with NAR for 24 h, then were digested with trypsin and were made into cell suspension, were washed with precooling PBS twice, were fixed with 75% precooling ethanol overnight at 4°C, collected the cells by centrifuge, were washed with 1 mL PBS, were added 100 *μ*L RNaseA incubated at 37°C for 30 min, were added 400 *μ*L PI incubated at 4°C for 30 min at dark, 1 × 10^4^ cells for cell cycle analysis with BD flow cytometry by standard procedure.

### 2.6. Let-7a Transfection Experiments

MMCs were seeded at a density of 0.75 × 10^6^ cells/mL in serum-free DMEM, with the addition of a transfection agent and let-7a mimics oligonucleotides (let7a-M), negative control mimics (NC-M), let-7a inhibitor oligonucleotides (let7a-I), and negative control inhibitor (NC-I) (Jima, Shanghai, China). Six hours after transfection, the medium was changed and the cells were incubated with fresh serum-containing medium for another 24~48 h. In addition, MMCs were transfected with let-7a mimics plus NAR as the NAR + let7a-M group; cells were transfected with let-7a inhibitor plus NAR as the NAR + let7a-I group. All transfections were performed with the aid of a lipofectamine 2000 transfection agent (Invitrogen, USA) following the manufacturer's instructions. Transfected cells were harvested at the indicated times for RNA and protein isolation.

### 2.7. Real-time RT PCR

Total RNA was extracted by Trizol reagent (Invitrogen) according to the manufacturer's instructions. Small RNAs (<300 nt) were isolated when 2 to 5 *μ*g total RNA sample was size-fractionated using a YM-100 Microcon centrifugal filter (Millipore). The PrimeScript RT reagent Kit (Takara, Dalian, China) and SYBR Premix Ex Taq TM II (Takara, Dalian, China) were used for miRNA and mRNA quantification. Relative expressions were calculated using 2^−ΔΔCT^ method and normalized to the expression of U6 or GAPDH. Primers for real-time PCR were as follows: Col-4, (F): 5′-CAAACCACAGCCAATCCTTCA-3′, (R): 5′-AAGAAGGGAAAACCCACTGTAGAGT-3′; FN, F: 5′-CATGGCTTTAGGCGAACCA-3′, R: 5′-CATCTACATTCGGCAGGTATGG-3′; TGF-*β*1, F: 5′-AGGGCTACCATGCCAACTTC-3′, R: 5′-CCACGTAGTAGACGATGGGC-3′; TGFBR1, F: 5′-TGGCGGAATCCACGAAGA-3′, R: 5′-ACGGATGGATCAGAAGGTACAAG-3′; Smad2, F: 5′-ACAACAGGCCTTTACAGCTTC-3′, R: 5′-CTCTGTGGCTCAATTCCTGC-3′; Smad7, F: 5′-CCATCAAGGCTTTTGACTATGAAA-3′, R: 5′-CCATGGCTGCTGCATGAAC-3′. All real-time RT PCR was performed in triplicate, and the data were presented as means ± SD.

### 2.8. Western Blotting

Total protein from cells and renal tissues were extracted, subjected to 10% SDS-polyacrylamide gels (Beyotime, Shanghai, China) and transferred to polyvinylidene difluoride membrane. Membranes were blocked with 5% nonfat dry milk dissolved in TBST and then incubated with primary antibody overnight at 4°C. Antibodies and dilutions included the following: rabbit monoclonal to Col4 antibody (1 : 1,000, Proteintech), rabbit polyclonal to FN antibody (1 : 1,000, Proteintech), rabbit monoclonal to smad2 antibody (1 : 1,500, Proteintech), rabbit polyclonal to p-smad2 antibody (1 : 500, Bioworld), rabbit polyclonal to TGF-*β*1 antibody (1 : 800, Proteintech), rabbit polyclonal to TGFBR1 antibody (1 : 700, Bioworld), and rabbit polyclonal to GAPDH antibody (1 : 2,000, Proteintech). The membranes were incubated with the goat anti-rabbit IgG (1 : 5,000, Abcam) for 1.5 h. Signals were detected with ChemiDocXRS system (Bio-Rad, USA). Grey values of protein bands were analyzed by Quantity One software.

### 2.9. Immunofluorescence

Kidney tissue paraffin sections (4 *μ*m) were subjected to immunofluorescence staining.Antibodies and dilutions were as follows: rabbit monoclonal to Col4 antibody (1 : 50, Proteintech), rabbit polyclonal to FN antibody (1 : 100, Proteintech), rabbit polyclonal to TGF-*β*1 antibody (1 : 50, Proteintech), and rabbit polyclonal to TGFBR1 antibody (1 : 50, Bioworld). The tissue sections were then incubated with FITC or TRITC for 1 h. Images were acquired using the fluorescence microscope (Olympus Japan).

### 2.10. 3′-UTR-Luciferase-Reporter Analysis

TGFBR1 (NM_009370.2) 3′-UTR-luciferase constructs were made using the pmiR-RB-REPORT*™* vector, and the primers for TGFBR1 3′ UTR-wt were as follows: TGFBR1-wt-F: 5′-GCGCTCGAGGGGTGTTTAGGAGGCTGGT-3′; TGFBR1-wt-R: 5′-AATGCGGCCGCCATACAACTTTTCCTTCGG-3′. The PCR products were excised with* NotI* and* XhoI*. The potential binding sequences of let-7a on the TGFBR1 3′UTR were mutated by the QuikChange*™* Site-Directed Mutagenesis Kit (Stratagene). For transfection, 293T cells were seeded into 96-well plates at a density of 1.5 × 10^4^ per well; then cells were transfected with TGFBR1-3′UTR-wt (200 ng/mL), TGFBR1-3′UTR-mut (200 ng/mL), and let-7a mimics (50 nmol/L) using lipofectamine*™* 2000. After 48 h, cells were lysed and assayed for luciferase activities. The ratio of firefly to* Renilla *activity was calculated. All experiments were performed in triplicate.

### 2.11. Statistical Analysis

All statistical tests were performed by SPSS19.0. Data for multiple variable comparisons were analyzed by one-way analysis of variance (ANOVA). Results were presented as means ± SD. Statistical significance was accepted at the 95% confidence level (*p* < 0.05).

## 3. Results

### 3.1. Effects of NAR on Blood Glucose Level and Renal Function and Morphology in DN Rats

**Figure 1:**
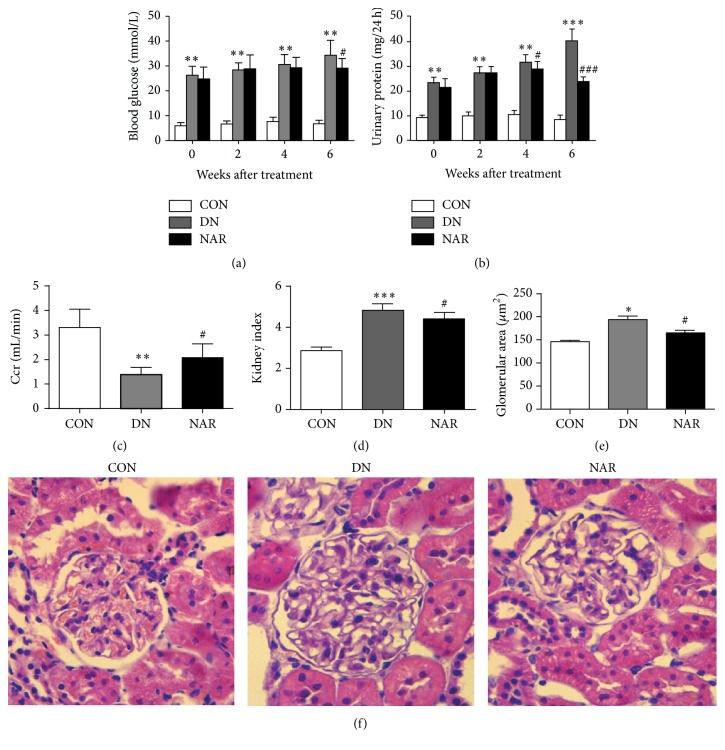
Effects of NAR on renal morphology and functions in diabetic rats. (a) Blood glucose level, (b) 24 h urinary protein, (c) urinary creatinine clearance ratio (Ccr), (d) kidney index (KI) which equals right kidney weight/body weight × 1000, (e) glomerular area (GA), and (f) representative photograph for hematoxylin-eosin (HE, ×400) are shown. Results are expressed as mean ± standard deviation, CON (*n* = 5), DN (*n* = 7), and NAR (*n* = 8). (a–d) ^*∗*^ *p* < 0.05, ^*∗∗*^ *p* < 0.01, and ^*∗∗∗*^ *p* < 0.001 versus CON group; ^#^ *p* < 0.05, ^##^ *p* < 0.01, and ^###^ *p* < 0.001 versus DN group.

Results showed blood glucose level went up steadily with time in DN rats, while blood glucose level was decreased at 6 weeks in NAR group (Figure 1(a)). The 24 h urinary protein went up steadily with time in DN rats, while after NAR treatment, it was decreased (Figure 1(b)). And creatinine clearance ratio (Ccr) is generally considered as the feature of renal function, reflecting renal filtration function. Kidney index (KI) and glomerular area (GA) are the morphological markers of glomerular hypertrophy. We evaluated the effects of NAR on 24 h urinary protein, Ccr, KI, and GA. 24 h urinary protein (8.49 ± 1.87 versus 40.41 ± 4.68 mg/24 h, *p* < 0.001). KI (2.87 ± 0.17 versus 4.83 ± 0.30, *p* < 0.001) and GA (146.32 ± 23.74 versus 194.29 ± 40.60 *μ*m^2^, *p* < 0.05) were significantly increased in DN group compared with those in CON group, whereas Ccr (3.31 ± 0.74 versus 1.39 ± 0.30 mL/min, *p* < 0.01) was decreased in DN group. After treatment with NAR, Ccr was obviously increased (1.39 ± 0.30 versus 2.08 ± 0.55 mL/min, *p* < 0.05) (Figure 1(c)); KI was significantly decreased (4.83 ± 0.30 versus 4.33 ± 0.34, *p* < 0.05) (Figure 1(d)). GA was reduced (194.29 ± 40.60 versus 165.15 ± 41.15 *μ*m^2^, *p* < 0.05) in the NAR group compared with that in DN group (Figures 1(e) and 1(f)). Together, our results suggested that NAR ameliorated renal structure and function.

### 3.2. Effects of NAR on Cell Proliferation, Cell Cycle, and Expressions of Col4 and FN

**Figure 2:**
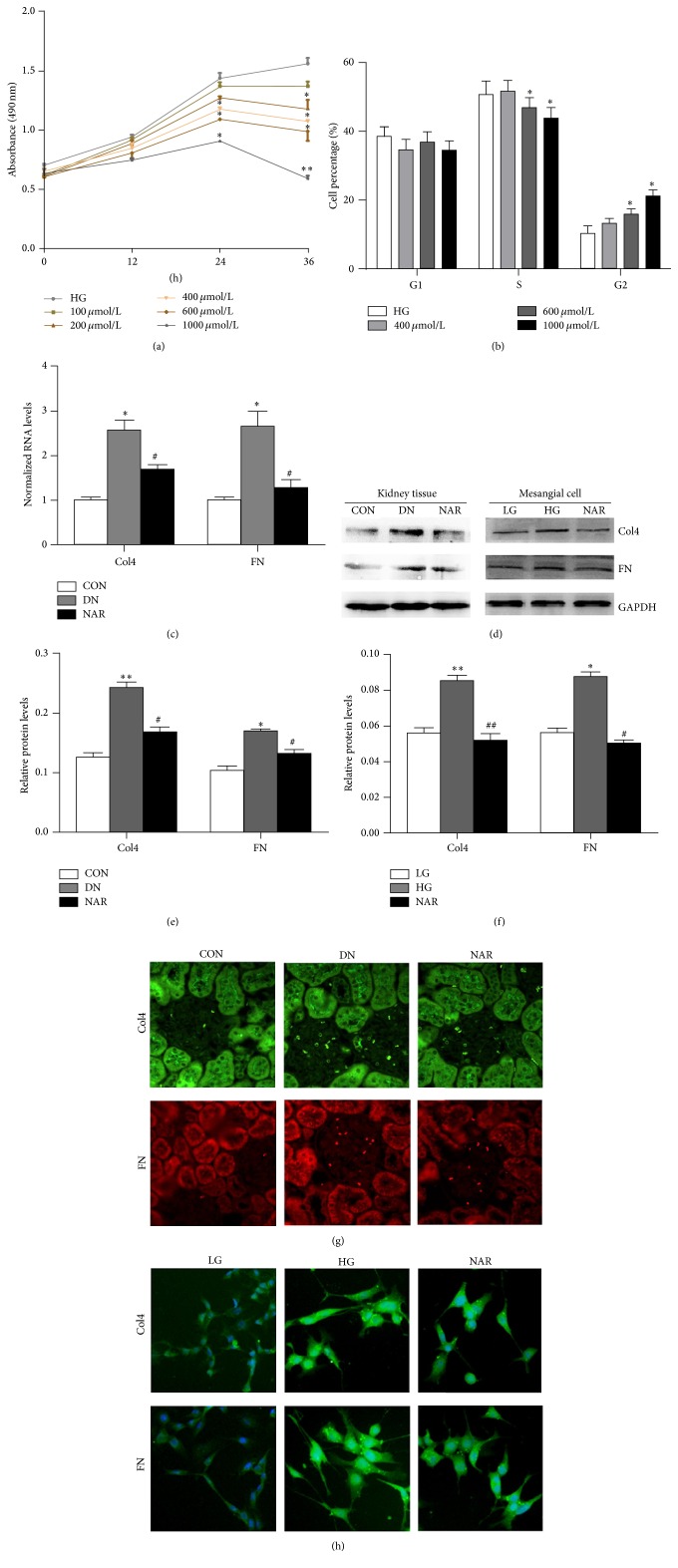
Effects of NAR on cell proliferation, cell cycle, and deposition of ECM proteins. (a) MTT analyses of cell proliferation rates. (b) Quantifications of the phases of the cell cycle analysis by flow cytometry. (c) Real-time RT PCR analyses of Col4 and FN mRNAs from kidney tissues. (d) Western blot analysis of Col4 and FN proteins in kidney tissues and MMCs. (e) Quantifications of the western blot bands of Col4 and FN expressions in DN rats. (f) Quantifications of the western blot bands of Col4 and FN expressions in MMCs. (g) Immunofluorescence of Col4 and FN proteins in kidney tissues of rats. (h) Immunofluorescence of Col4 and FN proteins in MMCs. (a, b) Results are expressed as mean ± standard deviation (*n* = 3 per group). ^*∗*^ *p* < 0.05; ^*∗∗*^ *p* < 0.01 versus HG group. (c) Results were normalized with GAPDH and expressed as mean ± standard deviation (*n* = 4 per group). ^*∗*^ *p* < 0.05 versus CON group; ^#^ *p* < 0.05 versus DN group. (e) Results were normalized with GAPDH and expressed as mean ± standard deviation (*n* = 3 per group). ^*∗*^ *p* < 0.05; ^*∗∗*^ *p* < 0.01 versus CON group; ^#^ *p* < 0.05 versus DN group. (f) Results were normalized with GAPDH and expressed as mean ± standard deviation (*n* = 3 per group). ^*∗*^ *p* < 0.05; ^*∗∗*^ *p* < 0.01 versus LG group; ^#^ *p* < 0.05; ^##^ *p* < 0.01 versus HG group.

Excessive mesangial cells proliferation increased the production of ECM. To evaluate the effect of NAR on mesangial cells proliferation, MTT and cell cycle analysis were used. MTT results showed that NAR obviously inhibited cell proliferation at the concentration of 400, 600, and 1000 *μ*mol/L for 24 h, 36 h, and 200 *μ*mol/L for 36 h, whereas cell proliferation rates were unchanged at the concentration of 100 *μ*mol/L (Figure 2(a)). Moreover, the cell cycle analysis showed cells were treated with 400, 600, and 1000 *μ*mol/L NAR for 24 h, respectively. The results showed that G2 phase of NAR group was significantly increased when compared with that in HG group, while S phase was shortened in NAR group at the concentration of 600 and 1000 *μ*mol/L. Therefore, these data indicated that NAR delayed mesangial cell growth at G2 phase (Figure 2(b)).

A key feature of DN was the accumulation of excessive extracellular matrix proteins, predominant collagens (Col4), and fibronectin (FN). To determine whether NAR affected Col4 and FN expression in vivo and in vitro, Col4 and FN mRNAs and proteins of kidney tissues were examined by real-time RT PCR (Figure 2(c)), western blot (Figures 2(d), 2(e), and 2(f)), and immunofluorescence (Figures 2(g) and 2(h)). The results showed that the mRNA and protein of Col4 and FN were remarkably increased in the DN or HG group compared with the CON or LG group and decreased when NAR was treated in mice and cells. Taken together, our results suggested that NAR could improve the excessive deposition of ECM.

### 3.3. Effect of NAR on Col4 and FN Expressions with Let-7a Intervention

**Figure 3:**
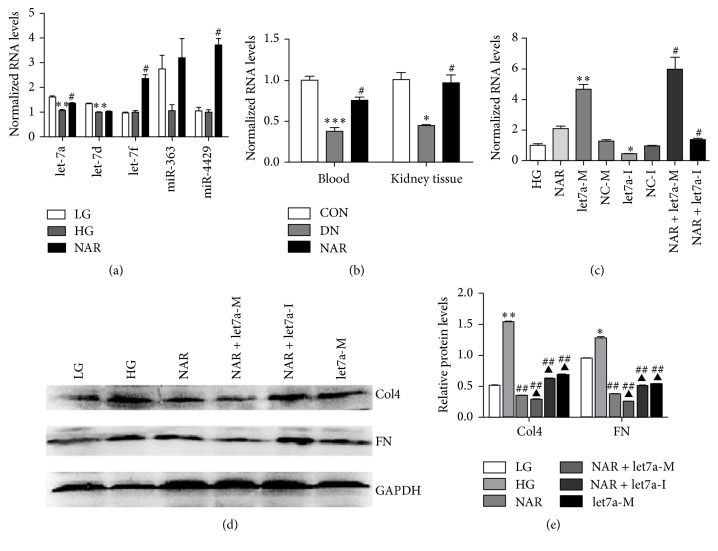
NAR inhibited deposition of ECM by upregulating let-7a expression. (a) MiRNAs expressions in MMCs treated with NAR. (b) Real-time RT PCR analysis of let-7a in blood and kidney tissues of rats. (c) Real-time RT PCR analysis of let-7a expressions. Data showed let-7a was upregulated or downregulated by let-7a mimics or let-7a inhibitor. Moreover, let-7a was increased in NAR group compared with HG untreated group. (d) Western blot analysis of Col4 and FN proteins in MMCs transferring let-7a mimics or inhibitor with NAR. (e) Quantifications of the western blot bands of Col4 and FN expressions in MMCs transferring let-7a mimics or inhibitor with NAR. (a) Results were normalized with U6 and expressed as mean ± standard deviation (*n* = 3 per group). ^*∗∗*^ *p* < 0.01 versus LG group; ^#^ *p* < 0.05 versus HG group. (b) Results were normalized with U6 and expressed as mean ± standard deviation (*n* = 4 per group). ^*∗*^ *p* < 0.05; ^*∗*^ *p* < 0.05; ^*∗∗∗*^ *p* < 0.001 versus CON group; ^#^ *p* < 0.05 versus DN group. (c) Results were normalized with U6 and expressed as mean ± standard deviation (*n* = 3 per group). ^*∗*^ *p* < 0.05; ^*∗∗*^ *p* < 0.01 versus HG group; ^#^ *p* < 0.05 versus NAR group. (e) Results were normalized with GAPDH and expressed as mean ± standard deviation (*n* = 3 per group). ^*∗∗*^ *p* < 0.01 versus LG group; ^##^ *p* < 0.01 versus HG group; ^▲^ *p* < 0.05 versus NAR group.

Our previous study demonstrated that several miRNAs were differentially expressed in blood samples of DN patients by miRNA array and real-time PCR, including let-7a, let-7d, let-7f, miR-363, and miR-4429 (data not shown). Furthermore, our result displayed that only let-7a was reversed when treated with NAR among the five miRNAs in mesangial cells with high glucose (Figure 3(a)). To determine whether NAR affected the expression of let-7a in vivo, let-7a expression was examined in blood and kidney tissue of DN rats. The results showed that let-7a was significantly decreased in the DN group compared with the CON group. After the treatment of NAR, the expression of let-7a was significantly enhanced in the NAR group compared with the DN group (Figure 3(b)). These results demonstrated that NAR upregulated let-7a expression both in vivo and in vitro in DN.

We further observed whether NAR affected fibrosis markers Col4 and FN expressions by regulating let-7a in cells. MMCs were transfected with let-7a mimics (let7a-M), let-7a inhibitor (let7a-I), NAR, or NAR with let-7a intervention. The results showed that let-7a was highly expressed in let-7a mimics group and decreased in inhibitor group. The expression of let-7a was significantly elevated in NAR + let-7a group, whereas let-7a declined in NAR + let-7a inhibitor group (Figure 3(c)). Moreover, the expressions of Col4 and FN were decreased in let-7a or NAR group compared with those in the untreated HG group. Interestingly, the expressions of Col4 and FN were lower after transferring let-7a mimic and NAR into mesangial cell compared with NAR or let-7a group (Figures 3(d) and 3(e)). So our results suggested that NAR could improve the excessive deposition of ECM by upregulating let-7a expression.

### 3.4. Let-7a Negatively Regulated TGFBR1 Expression

**Figure 4:**
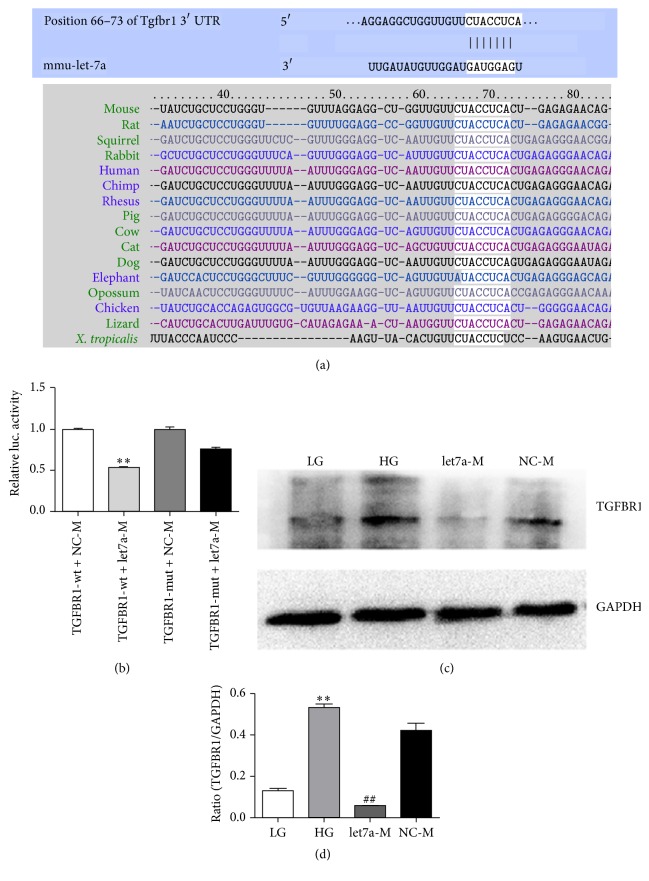
TGFBR1 was a target for let-7a. (a) Let-7a binding region of the 3′-UTR of TGFBR1 from fifteen species. Paired bases were exhibited by a black line. And the binding site was boxed with a black rectangle. (b) Luciferase activity of reporters in the presence or absence of let-7a mimics in 293T cells. Luc activity was significantly decreased in TGFBR1-wt+let7a-M. (c) Let-7a negatively regulated the expression of TGFBR1 in MMCs by western blot. (d) Quantifications of the western blot bands. (b) Data shown as mean ± standard deviation (*n* = 3 per group). ^*∗∗*^ *p* < 0.01 versus TGFBR1-wt+NC-M group. (d) Results were normalized with GAPDH and expressed as mean ± standard deviation (*n* = 3 per group). ^*∗∗*^ *p* < 0.01 versus LG group; ^##^ *p* < 0.01 versus HG group.

To evaluate possible functions of let-7a, we screened potential target genes of let-7a. Firstly, according to bioinformatics software Targetscan (http://www.targetscan.org/), we found that DN related gene-TGFBR1 was a potential target of let-7a. By analyzing homology, we found that the putative mmu-let-7a target site in TGFBR1 3′-UTR was highly conserved in sixteen genomes (Figure 4(a)). Secondly, we performed the dual-luciferase reporter assay and western blot. Transcripts carrying the TGFBR1 3′-UTR-wt exhibited a significant reduction in luciferase activity in the presence of let-7a. In contrast, the mimics negative control had no significant effect on the luciferase activity (Figure 4(b)). Meanwhile, western blot results showed that the expression level of TGFBR1 protein was reduced significantly in the let-7a mimics group compared with the HG group (Figures 4(c) and 4(d)). The data indicated that let-7a could negatively regulated TGFBR1 expression. Therefore, TGFBR1 was a target for let-7a.

### 3.5. Effect of NAR on TGF-*β*1/Smads Signaling with Let-7a Intervention

**Figure 5:**
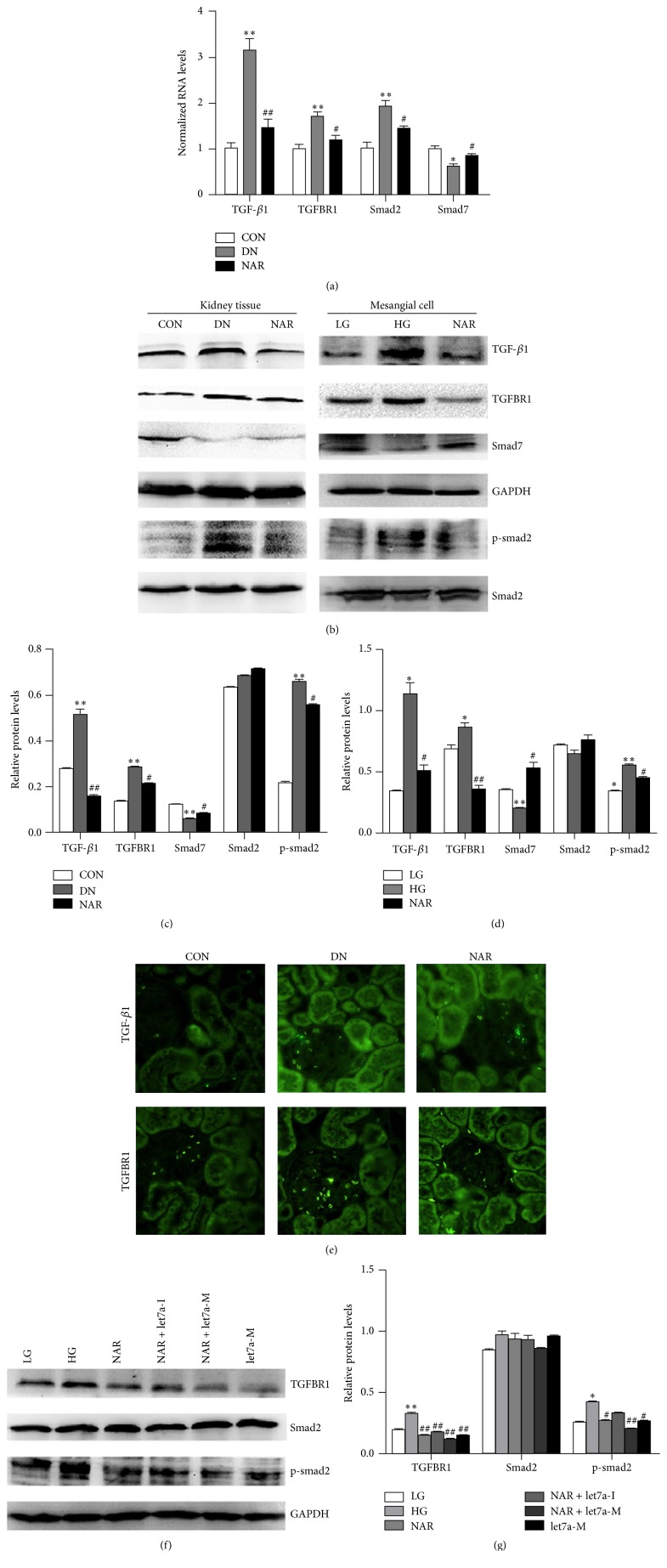
NAR inhibited TGF-*β*1/smads signaling through let-7a in vivo and in vitro. (a) Real-time RT PCR showed the mRNA levels of TGF-*β*1, TGFBR1, smad2, and smad7 of kidney tissues in rats. (b) Western blot analysis of TGF-*β*1, TGFBR1, smad2, p-smad2, and smad7 proteins in kidney tissues and MMCs. (c) Quantifications of the western blot bands of TGF-*β*1, TGFBR1, smad7, and p-smad2 expressions in DN rats. (d) Quantifications of the western blot bands of TGF-*β*1, TGFBR1, smad7, and p-smad2 expressions in MMCs. (e) Immunofluorescence of TGF-*β*1 and TGFBR1 proteins in kidney tissues of rats. (f) Western blot analysis of TGFBR1, smad2, and p-smad2 proteins in MMCs transferring let-7a mimics or inhibitor with NAR. (g) Quantifications of the western blot bands of TGFBR1 and p-smad2 expressions in MMCs transferring let-7a mimics or inhibitor with NAR. (a) Results were normalized with GAPDH and expressed as mean ± standard deviation (*n* = 4 per group). ^*∗*^ *p* < 0.05; ^*∗∗*^ *p* < 0.05 versus CON group; ^#^ *p* < 0.05; ^##^ *p* < 0.01 versus DN group. (c) Results were normalized with GAPDH and expressed as mean ± standard deviation (*n* = 3 per group). ^*∗∗*^ *p* < 0.01 versus CON group; ^#^ *p* < 0.05; ^##^ *p* < 0.01 versus DN group. (d) Results were normalized with GAPDH and expressed as mean ± standard deviation (*n* = 3 per group). ^*∗*^ *p* < 0.05; ^*∗∗*^ *p* < 0.01 versus LG group; ^#^ *p* < 0.05; ^##^ *p* < 0.01 versus HG group. (g) Results were normalized with GAPDH and expressed as mean ± standard deviation (*n* = 3 per group). ^*∗*^ *p* < 0.05; ^*∗∗*^ *p* < 0.01 versus LG group; ^#^ *p* < 0.05; ^##^ *p* < 0.01 versus HG group.

TGF-*β*1 is recognized as a major mediator of renal fibrosis because it is able to stimulate the accumulation of ECM protein and TGF-*β*1/smad signaling activation involving in this process [29]. To determine that whether NAR affected the TGF-*β*1/smad signaling in vivo, we examined TGF-*β*1, TGFBR1, smad2, and smad7 mRNA and protein expressions in kidney tissue of DN rats. The results of real-time RT PCR showed that TGF-*β*1, TGFBR1, and smad2 mRNAs were remarkably increased in the DN group compared with the CON group, while smad7 mRNA was declined. NAR reduced the expressions of TGF-*β*1, TGFBR1, and smad2 and increased smad7 mRNAs (Figure 5(a)). Meanwhile, the western blot results showed that TGF-*β*1/smad signaling was highly activated in DN group as revealed by a marked upregulation of TGF-*β*1, TGFBR1, and p-smad2/smad2 and downexpression of smad7, whereas NAR directly reduced TGF-*β*1, TGFBR1, and p-smad2/smad2 and increased smad7 protein expression (Figures 5(b) and 5(c)). We further observed the effects of NAR on TGF-*β*1, TGFBR1, smad2/p-smad2, and smad7 protein in MMCs. We found that the expressions of TGF-*β*1, TGFBR1, and smad2/p-smad2 significantly decreased and smad7 increased in NAR group compared with those in HG group (Figures 5(b) and 5(d)). The results of immunofluorescence for TGF-*β*1 and TGFBR1 were consistent with the results of western blot (Figure 5(e)). Together, our results suggested that TGF-*β*1/smad signaling was activated in DN rats and MMCs in high glucose condition and NAR could inhibit TGF-*β*1/smad signaling activation in vivo and vitro.

Furthermore, we observed whether NAR affected TGF-*β*1/smad signaling by regulating let-7a in MMCs or not. We found that overexpression of let-7a and NAR treatment decreased the ratio of p-smad2/smad2 and TGFBR1 proteins compared with MMCs without treatment. Additionally, NAR alleviated the change in the ratio of p-smad2/smad2 and TGFBR1 proteins cooperation with let7a-M more significantly than NAR group and let7a-M group, whereas the ratio of p-smad2/smad2 and TGFBR1 protein was increased in the NAR + let-7a inhibitor group compared with NAR group (Figures 5(f) and 5(g)). Our results suggested that TGF-*β*1/smads signaling was activated in DN rats and MMCs in high glucose condition and NAR inhibited TGF-*β*1/smads signaling activation through upregulating let-7a and suppressing TGFBR1 expression.

## 4. Discussion

DN is one of the most important diabetic microvascular complications. The mesangial cell proliferation plays an important role in DN. Glomerular mesangial cell acts actively in glomeruli, which can secrete ECM and produce cytokine, phagocyte, and clear macromolecules, and it has systolic function like smooth muscle cell. Studies have confirmed that mesangial cell was the important glomerular resident cells in the synthesis of ECM proteins [30, 31]. Deposition of excessive ECM molecules, predominant collagens, and fibronectin in the kidney will lead to kidney hyperplasia and glomerular area enlargement, which adversely affected the structure and function of the kidney. However, the underlying mechanism is not completely understood.

NAR is a natural flavonoid commonly occurring in many plant foods. It played an important role in diabetes and its complications [32]. Researches revealed that NAR could affect dyslipidemia, apoB overproduction, and hyperinsulinemia in LDL-receptor null mice with diet-induced insulin resistance [33]. NAR could play antihyperglycemic and antioxidative roles in diabetic rats [34]. And the antidiabetic effect of NAR might be insulin-like effect in type 2 diabetic rats [35]. Additionally, it was reported that NAR supplement increased its deposit in the liver and kidney of diabetic mice, and plasma levels of glucose and blood urea nitrogen were both decreased in NAR group, while insulin level and creatinine clearance were increased in NAR group compared with the diabetic control group [16]. In this study, our results showed NAR could not only decrease the blood glucose, 24 h urinary protein, kidney index, and glomerular area but also increase Ccr in DN rats. Also, we examined the therapeutic effects of NAR in DN rats and mesangial cells under diabetic condition. Our data showed that NAR inhibited mesangial cells proliferation by delaying cell cycle at G2 phase. Moreover, Col4 and FN were highly expressed in DN rats and MMCs under high glucose condition, while NAR directly reduced the expressions of Col4 and FN mRNA and proteins. Therefore, these findings displayed that NAR might participant in the development and progress of DN and suggested that NAR could play an important role in DN. However, the exact mechanism of NAR in DN is still unclear.

To explore the underlying mechanism of NAR in DN, several DN related miRNAs were focused on. Results showed let-7a was the only miRNA which reversed the expression level when treated with NAR in mesangial cells cultured with high glucose. Furthermore, numerous researches showed that let-7a was closely linked to modulate cell proliferation in various diseases [36–38]. Also, our previous studies also showed that the promoter hypermethylation and single nucleotide polymorphism of let-7a played important roles in DN [21, 22]. Therefore, these studies suggested that let-7a might be related to the therapeutic effects of NAR in DN. In our study, real-time RT PCR results exhibited that let-7a expression was significantly decreased in both blood and kidney tissues of DN rats and mesangial cells under hyperglycemic condition. And the expression of let-7a was elevated after NAR treatment in vivo and in vitro. These data suggested that NAR could upregulate the expression of let-7a in DN. Additionally, NAR alleviated the changes of Col4 and FN proteins cooperated with let7a mimics more significantly than NAR group, whereas the inhibitory effect of NAR cooperated with let-7a inhibitor was weakened. So these results suggested that NAR alleviated the deposition of ECM proteins by upregulating let-7a expression.

In the famous DN related pathway-TGF-*β*1/smad signaling pathway, the binding of TGF-*β*1 toTGFBR1 could activate phosphorylation of smad2 and smad3. Subsequently, phosphorylated smad2 and smad3 bound to the common smad4 and formed the smad complex to regulate the downstream gene transcription. In this process, smad7 could block the activation of TGF-*β*1/smad signaling as an inhibitory factor [39]. Therefore, TGFBR1, TGF-*β*1, and smads were key factors in the TGF-*β*1/smad signaling pathway. Research showed NAR decreased cell invasion and metastasis by inhibition of TGF-*β*1/smads signaling pathway in pancreatic cancer, and NAR also reduced TGF-*β*1-induced hepatic satellite cell extracellular matrix deposition by inhibiting TGF-*β*1/smads signals [12, 40]. In this study, our data displayed that there was a significant reduction of TGFBR1 protein in the presence of let-7a mimics by dual-luciferase reporter assay, and overexpression of let-7a could decrease the expression of TGFBR1 protein by western blot. These data verified that TGFBR1 was a target for let-7a.

Our results showed that TGF-*β*1/smads signaling was highly activated in DN rats and MMCs under high glucose condition, whereas NAR directly suppressed activation of TGF-*β*1/smad signaling. More importantly, in vitro experiments, overexpression of let-7a decreased the expression of p-smad2 and TGFBR1 proteins compared with HG group and it suggested that let-7a may negatively regulate TGF-*β*1/smads signaling by targeting TGFBR1. Meanwhile, NAR cooperated with let7a mimics; its effects were more remarkable than NAR or let-7a mimics' individual effect in the reductions of p-smad2 and TGFBR1 proteins, while the reductions of p-smad2 and TGFBR1 proteins in the NAR cooperated with let-7a inhibitor group were weakened. Taken together, our data exhibited that NAR regulated the alteration of TGF-*β*1/smads signaling through upregulating let-7a expression and suppressing TGFBR1.

In summary, our study suggested that NAR ameliorated kidney injury and inhibited mesangial cells proliferation and accumulation of ECM in DN. NAR played a biological role in TGF-*β*1/smad signaling pathway by regulating let-7a/TGFBR1, and let-7a might be a novel therapeutic target for NAR protect against DN.

## References

[1] Sharma K., Ziyadeh F. N. (1995). Hyperglycemia and diabetic kidney disease. The case for transforming growth factor-*β* as a key mediator. *Diabetes*.

[2] Forbes J. M., Coughlan M. T., Cooper M. E. (2008). Oxidative stress as a major culprit in kidney disease in diabetes. *Diabetes*.

[3] Piotrowski D. W. (2012). Mineralocorticoid receptor antagonists for the treatment of hypertension and diabetic nephropathy. *Journal of Medicinal Chemistry*.

[4] Wada J., Makino H. (2013). Inflammation and the pathogenesis of diabetic nephropathy. *Clinical Science*.

[5] Al-Rejaie S. S., Aleisa A. M., Abuohashish H. M. (2015). Naringenin neutralises oxidative stress and nerve growth factor discrepancy in experimental diabetic neuropathy. *Neurological Research*.

[6] Banjerdpongchai R., Wudtiwai B., Khaw-on P., Rachakhom W., Duangnil N., Kongtawelert P. (2016). Hesperidin from Citrus seed induces human hepatocellular carcinoma HepG2 cell apoptosis via both mitochondrial and death receptor pathways. *Tumor Biology*.

[7] Martinez R. M., Pinho-Ribeiro F. A., Steffen V. S. (2015). Naringenin inhibits UVB irradiation-induced inflammation and oxidative stress in the skin of hairless mice. *Journal of Natural Products*.

[8] Orhan I. E., Nabavi S. F., Daglia M., Tenore G. C., Mansouri K., Nabavi S. M. (2015). Naringenin and atherosclerosis: a review of literature. *Current Pharmaceutical Biotechnology*.

[9] Bodduluru L. N., Kasala E. R., Madhana R. M. (2016). Naringenin ameliorates inflammation and cell proliferation in benzo(a)pyrene induced pulmonary carcinogenesis by modulating CYP1A1, NF*κ*B and PCNA expression. *International Immunopharmacology*.

[10] Song H. M., Park G. H., Eo H. J. (2015). Anti-proliferative effect of naringenin through p38-dependent downregulation of cyclin D1 in human colorectal cancer cells. *Biomolecules and Therapeutics*.

[11] Li R.-F., Feng Y.-Q., Chen J.-H., Ge L.-T., Xiao S.-Y., Zuo X.-L. (2015). Naringenin suppresses K562 human leukemia cell proliferation and ameliorates Adriamycin-induced oxidative damage in polymorphonuclear leukocytes. *Experimental and Therapeutic Medicine*.

[12] Liu X., Wang W., Hu H. (2006). Smad3 specific inhibitor, naringenin, decreases the expression of extracellular matrix induced by TGF-*β*1 in cultured rat hepatic stellate cells. *Pharmaceutical Research*.

[13] Sánchez-Salgado J. C., Ortiz-Andrade R. R., Aguirre-Crespo F. (2007). Hypoglycemic, vasorelaxant and hepatoprotective effects of Cochlospermum vitifolium (Willd.) Sprengel: a potential agent for the treatment of metabolic syndrome. *Journal of Ethnopharmacology*.

[14] Priscilla D. H., Roy D., Suresh A., Kumar V., Thirumurugan K. (2014). Naringenin inhibits *α*-glucosidase activity: a promising strategy for the regulation of postprandial hyperglycemia in high fat diet fed streptozotocin induced diabetic rats. *Chemico-Biological Interactions*.

[15] Yoshida H., Watanabe H., Ishida A. (2014). Naringenin suppresses macrophage infiltration into adipose tissue in an early phase of high-fat diet-induced obesity. *Biochemical and Biophysical Research Communications*.

[16] Tsai S.-J., Huang C.-S., Mong M.-C., Kam W.-Y., Huang H.-Y., Yin M.-C. (2012). Anti-inflammatory and antifibrotic effects of naringenin in diabetic mice. *Journal of Agricultural and Food Chemistry*.

[17] Frost R. J. A., Olson E. N. (2011). Control of glucose homeostasis and insulin sensitivity by the Let-7 family of microRNAs. *Proceedings of the National Academy of Sciences of the United States of America*.

[18] Zhu H., Shyh-Chang N., Segr A. V. (2011). The *Lin28/let-7* axis regulates glucose metabolism. *Cell*.

[19] Jiang L. Q., Franck N., Egan B. (2013). Autocrine role of interleukin-13 on skeletal muscle glucose metabolism in type 2 diabetic patients involves microRNA let-7. *The American Journal of Physiology—Endocrinology and Metabolism*.

[20] Park J. T., Kato M., Lanting L. (2014). Repression of let-7 by transforming growth factor-*β*1-induced Lin28 upregulates collagen expression in glomerular mesangial cells under diabetic conditions. *American Journal of Physiology-Renal Physiology*.

[21] Peng R., Liu H., Peng H. (2015). Promoter hypermethylation of let-7a-3 is relevant to its down-expression in diabetic nephropathy by targeting UHRF1. *Gene*.

[22] Zhou J., Peng R., Li T. (2013). A potentially functional polymorphism in the regulatory region of let-7a-2 is associated with an increased risk for diabetic nephropathy. *Gene*.

[23] Dangi-Garimella S., Strouch M. J., Grippo P. J., Bentrem D. J., Munshi H. G. (2011). Collagen regulation of let-7 in pancreatic cancer involves TGF-*β*1-mediated membrane type 1-matrix metalloproteinase expression. *Oncogene*.

[24] Liu Y., Li H., Feng J. (2013). Lin28 induces epithelial-to-mesenchymal transition and stemness via downregulation of let-7a in breast cancer cells. *PLoS ONE*.

[25] Makino K., Jinnin M., Hirano A. (2013). The downregulation of microRNA let-7a contributes to the excessive expression of type I collagen in systemic and localized scleroderma. *Journal of Immunology*.

[26] Srinivasan K., Viswanad B., Asrat L., Kaul C. L., Ramarao P. (2005). Combination of high-fat diet-fed and low-dose streptozotocin-treated rat: a model for type 2 diabetes and pharmacological screening. *Pharmacological Research*.

[27] Zhao T. T., Zhang H. J., Lu X. G. (2014). Chaihuang-Yishen granule inhibits diabetic kidney disease in rats through blocking TGF-*β*/Smad3 signaling. *PLoS ONE*.

[28] Yokozawa T., Nakagawa T., Wakaki K., Koizumi F. (2001). Animal model of diabetic nephropathy. *Experimental and Toxicologic Pathology*.

[29] Lan H. Y. (2012). Transforming growth factor-*β*/Smad signalling in diabetic nephropathy. *Clinical and Experimental Pharmacology & Physiology*.

[30] Nerlich A., Schleicher E. (1991). Immunohistochemical localization of extracellular matrix components in human diabetic glomerular lesions. *The American Journal of Pathology*.

[31] Floege J., Johnson R. J., Gordon K. (1991). Increased synthesis of extracellular matrix in mesangial proliferative nephritis. *Kidney International*.

[32] Chen J., Mangelinckx S., Adams A., Wang Z.-T., Li W.-L., De Kimpe N. (2015). Natural flavonoids as potential herbal medication for the treatment of diabetes mellitus and its complications. *Natural Product Communications*.

[33] Mulvihill E. E., Allister E. M., Sutherland B. G. (2009). Naringenin prevents dyslipidemia, apolipoprotein B overproduction, and hyperinsulinemia in LDL receptor-null mice with diet-induced insulin resistance. *Diabetes*.

[34] Annadurai T., Muralidharan A. R., Joseph T., Hsu M. J., Thomas P. A., Geraldine P. (2012). Antihyperglycemic and antioxidant effects of a flavanone, naringenin, in streptozotocin-nicotinamide-induced experimental diabetic rats. *Journal of Physiology and Biochemistry*.

[35] Ortiz-Andrade R. R., Sánchez-Salgado J. C., Navarrete-Vázquez G. (2008). Antidiabetic and toxicological evaluations of naringenin in normoglycaemic and NIDDM rat models and its implications on extra-pancreatic glucose regulation. *Diabetes, Obesity & Metabolism*.

[36] Tang R., Yang C., Ma X. (2016). MiR-let-7a inhibits cell proliferation, migration, and invasion by down-regulating PKM2 in gastric cancer. *Oncotarget*.

[37] Li Y., Zhang X., Chen D., Ma C. (2016). Let-7a suppresses glioma cell proliferation and invasion through TGF-*β*/Smad3 signaling pathway by targeting HMGA2. *Tumor Biology*.

[38] He X.-Y., Chen J.-X., Ou-Yang X., Zhang Z., Peng H.-M. (2010). Construction of let-7a expression plasmid and its inhibitory effect on k-Ras protein in A549 lung cancer cells. *Journal of Southern Medical University*.

[39] Chen H. Y., Huang X. R., Wang W. (2011). The protective role of Smad7 in diabetic kidney disease: mechanism and therapeutic potential. *Diabetes*.

[40] Lou C., Zhang F., Yang M. (2012). Naringenin decreases invasiveness and metastasis by inhibiting TGF-*β*-induced epithelial to mesenchymal transition in pancreatic cancer cells. *PLoS ONE*.

